# Supplementary material for: Transgenerational effects of grandparental and parental diets combine with early-life learning to shape adaptive foraging phenotypes in Amblyseius swirskii
Source: Commun Biol. 2022 Mar 21;5:246. doi: 10.1038/s42003-022-03200-7 (PMC8938427; doi:10.1038/s42003-022-03200-7)
Supplement: Supplementary file 2 — Supplementary Information (new) [file 42003_2022_3200_MOESM2_ESM.docx]

**Supplementary Material for**

**Transgenerational effects of grandparental and parental diets combine with early-life learning to shape adaptive foraging phenotypes in mites**

Peter Schausberger^1,*^ & Dalila Rendon^1^

^1^Department of Behavioral and Cognitive Biology, University of Vienna, Djerassiplatz 1, 1030 Vienna, Austria

*Corresponding author: [peter.schausberger@univie.ac.at](mailto:peter.schausberger@univie.ac.at)

This file contains information on model selection and estimated marginal means in statistical analyses (six supplementary tables)

**Supplementary Note 1**

Software used: IBM SPSS Statistics version 28.0

Model selection started with the full model and removed stepwise non-significant 3-way and 2-way interactions until arriving at the most parsimonious model (difference >2 to the next based model) based on the Bayesian Information Criterion (BIC) for generalized linear models (GLM) and Quasi Likelihood Information Criterion (QIC) for generalized estimating equations (GEE).

Estimated marginal means are given for significant (*P* < 0.05) main and interaction effects; if a significant main effect interacted significantly with another main effect, only the marginal means of the interaction effect are given (see also tables 1 and 2 in the paper).

**Supplementary Table 1.** Attack latency on 1^st^ thrips (min; log transformed); GLM results are in table 1, data visualization in figure 2**;** independent variables in GLM are grandparental diet, parental diet, and thrips experience in early life

Model selection (GLM) BIC

Full model 161.191

Non-significant 3-way interaction removed 153.747

Non-significant 2- and 3-way interactions removed 131.190

Estimated marginal means for grandparental diet and thrips experience

| Grandparental Diet | Mean | Std. Error |
| --- | --- | --- |
|  |  |  |
| Pollen | 1.7647 | .03134 |
| Live Spider Mites | 1.6722 | .02955 |

| Thrips Experience | Mean | Std. Error |
| --- | --- | --- |
|  |  |  |
| No | 1.8276 | .03071 |
| Yes | 1.6093 | .03014 |

**Supplementary Table 2. Eggs per female per 24h;** GLM results are in table 1, data visualization in figure 6**;** independent variables in GLM are grandparental diet, parental diet, and thrips experience in early life

Model selection (GLM) BIC

Full model 515.544

Non-significant 3-way interaction removed 505.243

Non-significant 2- and 3-way interactions removed 499.971

Estimated marginal means for the interaction grandparental diet*parental diet

| Grandparental Diet | Parental Diet | Mean | Std. Error |
| --- | --- | --- | --- |
|  |  |  |  |
| Pollen | Pollen | .84 | .131 |
|  | Live Spider Mites | .53 | .109 |
|  | Dead Spider Mites | .42 | .104 |
| Live Spider Mites | Pollen | 1.22 | .163 |
|  | Live Spider Mites | .27 | .075 |
|  | Dead Spider Mites | .20 | .062 |

**Supplementary Table 3.** Killed thrips per female per 24h; GLM results are in table 1, data visualization in figure 5; independent variables GLM are grandparental diet, parental diet, and thrips experience in early life

Model selection (GLM) BIC

Full model 792.227

Non-significant 3-way interaction removed 781.299

Non-significant 2- and 3-way interactions removed 764.971

Estimated marginal means for the interaction parental diet*thrips experience

| Parental Diet | Thrips Experience | Mean | Std. Error |
| --- | --- | --- | --- |
|  |  |  |  |
| Pollen | No | 2.51 | .133 |
|  | Yes | 2.75 | .074 |
| Live Spider Mites | No | 2.63 | .102 |
|  | Yes | 2.71 | .082 |
| Dead Spider Mites | No | 2.85 | .065 |
|  | Yes | 2.66 | .078 |

Estimated marginal means for the interaction parental diet*thrips experience

| Parental Diet | Thrips Experience | Mean | Std. Error |
| --- | --- | --- | --- |
|  |  |  |  |
| Pollen | No | .86 | .143 |
|  | Yes | 1.19 | .148 |
| Live Spider Mites | No | .29 | .088 |
|  | Yes | .49 | .092 |
| Dead Spider Mites | No | .51 | .102 |
|  | Yes | .16 | .062 |

**Supplementary Table 4.** Attack latencies on all three thrips (min; log transformed); GEE results are in table 1, data visualization in figure 3; independent variables in GEE are grandparental diet, parental diet, and thrips experience in early life

Model selection (GEE) QIC

Full model 233.176

Non-significant 3-way interaction removed 229.801

Non-significant 2- and 3-way interactions removed 224.911

Estimated marginal means for the interaction grandparental diet*parental diet

| Grandparental Diet | Parental Diet | Mean | Std. Error |
| --- | --- | --- | --- |
|  |  |  |  |
| Pollen | Pollen | 2.4035 | .03445 |
|  | Live Spider Mites | 2.3600 | .03226 |
|  | Dead Spider Mites | 2.2885 | .03536 |
| Live Spider Mites | Pollen | 2.1717 | .03118 |
|  | Live Spider Mites | 2.3501 | .03345 |
|  | Dead Spider Mites | 2.3149 | .03186 |

**Supplementary Table 5.** Number of thrips killed over time; GEE results are in table 2, data visualization in figure 4**;** independent variables in GEE are grandparental diet, parental diet, and thrips experience in early life

Model selection (GEE) QIC

Full model 621.233

Non-significant 3-way interaction removed 618.843

Non-significant 2- and 3-way interactions removed 613.606

Estimated marginal means for thrips experience

| Thrips Experience | Mean | Std. Error |
| --- | --- | --- |
|  |  |  |
| No | .92 | .063 |
| Yes | 1.15 | .070 |

**Supplementary Table 6.** Body size (dorsal shield area; mm²); GLM results are in the results section; independent variables in GLM are grandparental and parental diet

Model selection (GLM) BIC

Full model 1216.901

Non-significant 2-way interaction removed 1207.978

Estimated marginal means for grandparental and parental diet

| Grandparental Diet | Mean | Std. Error |
| --- | --- | --- |
|  |  |  |
| Pollen | 0.07937 | 0.00092 |
| Spider Mites | 0.07826 | 0.00087 |

| Parental Diet | Mean | Std. Error |
| --- | --- | --- |
|  |  |  |
| Pollen | 0.07873 | 0.00109 |
| Live TSSM | 0.07954 | 0.00098 |
| Dead TSSM | 0.07817 | 0.00123 |
