# Supplementary material for: Transgenerational effects of grandparental and parental diets combine with early-life learning to shape adaptive foraging phenotypes in Amblyseius swirskii
Source: Commun Biol. 2022 Mar 21;5:246. doi: 10.1038/s42003-022-03200-7 (PMC8938427; doi:10.1038/s42003-022-03200-7)
Supplement: Supplementary file 3 — Reporting Summary [file 42003_2022_3200_MOESM3_ESM.pdf]

nature portfolio

Corresponding author(s): Peter Schausberger

Last updated by author(s): Feb 1, 2022

Reporting Summary

Nature Portfolio wishes to improve the reproducibility of the work that we publish. This form provides structure for consistency and transparency in reporting. For further information on Nature Portfolio policies, see our Editorial Policies and the Editorial Policy Checklist.

Statistics

For all statistical analyses, confirm that the following items are present in the figure legend, table legend, main text, or Methods section.

n/a	Confirmed	
☐	☒	The exact sample size (*n*) for each experimental group/condition, given as a discrete number and unit of measurement
☐	☒	A statement on whether measurements were taken from distinct samples or whether the same sample was measured repeatedly
☐	☒	The statistical test(s) used AND whether they are one- or two-sided<br>*Only common tests should be described solely by name; describe more complex techniques in the Methods section.*
☒	☐	A description of all covariates tested
☐	☒	A description of any assumptions or corrections, such as tests of normality and adjustment for multiple comparisons
☐	☒	A full description of the statistical parameters including central tendency (e.g. means) or other basic estimates (e.g. regression coefficient) AND variation (e.g. standard deviation) or associated estimates of uncertainty (e.g. confidence intervals)
☐	☒	For null hypothesis testing, the test statistic (e.g. *F*, *t*, *r*) with confidence intervals, effect sizes, degrees of freedom and *P* value noted<br>*Give P values as exact values whenever suitable.*
☒	☐	For Bayesian analysis, information on the choice of priors and Markov chain Monte Carlo settings
☒	☐	For hierarchical and complex designs, identification of the appropriate level for tests and full reporting of outcomes
☒	☐	Estimates of effect sizes (e.g. Cohen's *d*, Pearson's *r*), indicating how they were calculated

Our web collection on statistics for biologists contains articles on many of the points above.

Software and code

Policy information about availability of computer code

Data collection	No software was used for data collection
Data analysis	The data were analyzed with IBM SPSS Statistics Version 28

For manuscripts utilizing custom algorithms or software that are central to the research but not yet described in published literature, software must be made available to editors and reviewers. We strongly encourage code deposition in a community repository (e.g. GitHub). See the Nature Portfolio guidelines for submitting code & software for further information.

Data

Policy information about availability of data

All manuscripts must include a data availability statement. This statement should provide the following information, where applicable:

- Accession codes, unique identifiers, or web links for publicly available datasets
- A description of any restrictions on data availability
- For clinical datasets or third party data, please ensure that the statement adheres to our policy

The raw data underlying the current study are available in figshare (https://doi.org/10.6084/m9.figshare.19243869).

Field-specific reporting

Please select the one below that is the best fit for your research. If you are not sure, read the appropriate sections before making your selection.

☐ Life sciences ☒ Behavioural & social sciences ☐ Ecological, evolutionary & environmental sciences

For a reference copy of the document with all sections, see nature.com/documents/nr-reporting-summary-flat.pdf

Behavioural & social sciences study design

All studies must disclose on these points even when the disclosure is negative.

Study description	Quantitative manipulative experiments on the behavioral ecology of invertebrate animals (mites)
Research sample	The samples range from 16 to 23 replicates per treatment for the behavioral test and 10 to 22 replicates for the body size measurements
Sampling strategy	Sampling was random, the required number of replicates was chosen based on >20 years experience with similar experiments and data.
Data collection	Quantitative data were collected by direct observation of the experimental animals using a Leica M80 stereo microscope. The experiments were partly blinded, that is, different persons prepared the experimental units with prey and others conducted the behavioral observations. Several persons were present in the laboratory during data collection.
Timing	All treatments were run in parallel.
Data exclusions	No data were excluded from analyses
Non-participation	Not applicable because this is a study on the behavioral ecology of invertebrate animals.
Randomization	Animals were randomly withdrawn from the stock population.

Reporting for specific materials, systems and methods

We require information from authors about some types of materials, experimental systems and methods used in many studies. Here, indicate whether each material, system or method listed is relevant to your study. If you are not sure if a list item applies to your research, read the appropriate section before selecting a response.

Materials & experimental systems

n/a	Involved in the study
☒	☐ Antibodies
☒	☐ Eukaryotic cell lines
☒	☐ Palaeontology and archaeology
☐	☒ Animals and other organisms
☒	☐ Human research participants
☒	☐ Clinical data
☒	☐ Dual use research of concern

Methods

n/a	Involved in the study
☒	☐ ChIP-seq
☒	☐ Flow cytometry
☒	☐ MRI-based neuroimaging

Animals and other organisms

Policy information about studies involving animals; ARRIVE guidelines recommended for reporting animal research

Laboratory animals	Experimental animals (mites) were derived from a laboratory-reared population founded with specimens collected in the field
Wild animals	Not applicable
Field-collected samples	The experimental animals were derived from a laboratory-reared population founded with animals collected in the field
Ethics oversight	No ethical approval was required (invertebrate animals that are neither protect nor endangered)

Note that full information on the approval of the study protocol must also be provided in the manuscript.
